# Gastrointestinal and urinary complaints in adults with hereditary spastic paraparesis

**DOI:** 10.1186/s13023-018-0804-8

**Published:** 2018-04-16

**Authors:** Øivind J. Kanavin, Krister W. Fjermestad

**Affiliations:** 1 Frambu Centre for Rare Disorders, Sandbakkveien 18, 1404 Siggerud, Norway; 2 Frambu Department of Psychology, University of Oslo, Oslo, Norway

**Keywords:** Hereditary spastic paraplegia, Fecal incontinence, Gastrointestinal complaints, Urinary incontinence, Urge incontinence

## Abstract

**Background:**

Hereditary spastic paraparesis (HSP) is a group of rare genetic disorders affecting the central nervous system. Pure HSP is limited to lower limb spasticity and urinary voiding dysfunction. Complex HSP involves additional neurological features. Beyond the described core symptoms, knowledge about the burden of disease for adults with HSP is limited, particularly regarding gastrointestinal functions, fecal incontinence, and urinary symptoms.

**Methods:**

We conducted a cross-sectional self-report survey with 108 adult HSP patients (M_age_ = 57.7 years, SD = 11.5, range 30 to 81; 54.2% females) recruited from a national HSP user group association and a national (non-clinical) advisory unit for rare disorders. HSP data was compared to data from a Norwegian population study, HUNT-3 (*N* = 46,293).

**Results:**

The HSP group reported more gastrointestinal and urinary complaints compared to controls. Gastrointestinal complaints included at least “*much*” complaints with constipation (14.6%) and alternating constipation/diarrhea (8.0%), and at least daily uncontrollable flatulence (47.6%), fecal incontinence (11.6%), and inability to hold back stools (38.5%). Urinary complaints included frequent urination (27.4% > 8 times daily), sudden urge (51.9%) and urinary incontinence (30.5% at least daily/nightly).

**Conclusion:**

This survey of adults with HSP recruited from non-clinical settings showed constipation, alternate constipation and diarrhea, fecal incontinence, and voiding dysfunction represent considerable problems for many persons with HSP. Health care providers should screen and manage often unrecognized gastrointestinal and fecal incontinence complaints among HSP patients.

## Background

The term hereditary spastic paraparesis (HSP) is used to describe a genetically and clinically heterogeneous group of neurodegenerative diseases [1]. Pure forms of HSP affect the lower limbs and lead to slowly progressing spastic weakness, and micturition dysfunction [2]. Complex HSP is not limited to the lower extremities, and may include additional neurological symptoms such as ataxia, extrapyramidal signs, epilepsy, mental retardation, dementia, and peripheral nerve involvement [1, 3]. Late onset HSP more often leads to rapid progression of symptoms, while early disease debut often heralds slower progression [2]. Several modes of inheritance are described, i.e., autosomal recessive, autosomal dominant, and more rarely, X-linked or maternal [4]. Complicated forms of HSP are mostly inherited in an autosomal recessive manner, while pure HSP is commonly inherited in an autosomal dominant manner [4]. At least 79 loci and 60 spastic paraplegia genes are currently identified [5]. In Norway, the site of the current study, the estimated combined prevalence of pure and complex HSP has been reported to be 7.4:100000 [6].

Increased voiding frequency, stress, and urge incontinence linked to neurogenic detrusor over activity has been described in adults with HSP [7–9]. It is less known to what extent fecal incontinence (FI), defined by unintentional loss of solid or liquid stool, is present in the HSP population. This is unfortunate, and in our experience, HSP patients regard FI as an important issue to address. The emphasis on FI symptoms was the result of close co-operation with representatives from the HSP patient user group in Norway, who experience reluctance among their members to discuss FI issues with health care providers. Despite lack of optimal treatment solutions for FI, some identified causal factors may be reversed by simple means such as diet, bowel habits, and toilet access [10].

Studies of non-HSP populations have shown FI is rarely disclosed to caregivers due to its’ sensitive nature and the stigma attached, and that FI can severely affect quality of life [10–12]. Four studies have examined gastrointestinal complaints in HSP populations. Anal sphincter dysfunction has been reported in HSP [13]. In a Dutch study of 18 HSP patients with a mutation in the *SPG11 gene,* (SPG11) eight were reported to have developed urinary and fecal incontinence [14]. In a study of 11 adult patients with autosomal dominant pure spastic paraplegia linked to chromosome 2p, five patients had symptoms of FI in addition to urinary incontinence and sexual dysfunction [15]. Recently, in a cohort of 118 HSP patients aged 10 to 84 years recruited from British neurological clinics, 26% of patients carrying mutations in the *SPAST gene* associated with SPG4 reported neurogenic bowel symptoms [16].

There have been few studies of the HSP population. Therefore, we conducted a large self-report survey of persons with HSP recruited from non-clinical settings, to examine the prevalence of gastrointestinal and urinary problems in the general HSP population. As symptoms of incontinence increase with age, we included persons aged 30 years and older. Our main research question was: What is the frequency of gastrointestinal complaints, urinary and fecal incontinence among persons with HSP compared to population controls?

## Method

### Sample and procedures

#### HSP sample

A total of 108 persons with HSP were recruited from two sources. The majority (61%) was recruited from the register of The National Patients’ Association for Hereditary Ataxia and Spastic Paraplegia (NASPA) in Norway. The remaining sample (39%) was recruited from the database of Frambu Resource Centre for Rare Disorders (Frambu). Frambu is one of nine state-financed centers of expertise administrated by the Norwegian National Advisory Unit on Rare Disorders. Registration in Frambu’s patient database is voluntary, based on patient consent, and does not require medical referral. Diagnostic confirmation from a medical institution is required to be registered. Initially we sent out questionnaires to the 60 persons with HSP aged 30 years or older registered in Frambu’s database. The questionnaire was also mailed to 235 NASPA members.

#### Control sample

Population data was drawn from the third follow up of the Norwegian epidemiological survey “Helseundersøkelsen i Nord-Trøndelag” (HUNT3) and used as controls [17]. HUNT3 is a comprehensive survey of health. All inhabitants in Nord-Trøndelag County aged 19 years and older were mailed questionnaires and invited to a clinical examination on three different occasions. HUNT3 (2006–2008) comprises the latest population data, providing recent results for gender and age groups comparable to our respondents. The control group comprised 46,293 persons, i.e., all participants aged 30 years and older from HUNT3. The study was planned and performed in cooperation with NASPA and approved by The Regional Committee for Medical and Health Research Ethics (REK South-East).

### Measures

The current survey was part of a larger study covering several areas of functioning [18]. The current report is based on 19 items, all drawn from HUNT3 [17]. Ten items covering urinary symptoms (e.g., frequency of daytime/nighttime urination) were derived from the Epidemiology of Incontinence in the County of Nord-Trøndelag (EPICONT), and The International Prostate Symptom Score (IPSS) [19]. Six items covering gastrointestinal problems (e.g., frequency of diarrhea, constipation) and three items covering bowel symptoms (e.g, frequency of FI) were developed for the HUNT study [17]. The items covered frequencies, experienced problems, and various details such as amount of leakage and attempted treatments. The questionnaire is available from the first author upon request.

### Data analysis

Data were analyzed using IBM SPSS version 21.0. Comparisons between samples were done by calculating Pearson’s chi-square differences for categorical data and Cohen’s *d*-effect size differences for scaled data.

## Results

### Response rate

The response rate from patients in the Frambu database was 80%. The exact response rate from the NASPA register is not known, as NASPA also has members with hereditary ataxias. The response rate in HUNT3 was 54%.

### Comparison of samples

The mean age between the HSP sample and controls was comparable (*M*_HSP_ = 57.7 years, *SD* = 11.5 vs. *M*_controls_ = 55.9 years, *SD* = 14.0, *d* = 0.13). The gender distribution between the HSP sample and controls was not significantly different (female ratio_HSP_ = 53.7% vs. female ratio_controls_ = 54.2%; (χ2 (1) = 0.011, *p* = .916). Within the HSP sample, participants recruited from NASPA were significantly older than participants recruited from Frambu (*M*_NASPA_ = 59.7 years, *SD* = 11.5 vs.*M*_Frambu_ = 54.6 years, *SD* = 10.9, *d* = 0.46). The gender distribution was not significantly different between recruitment settings (female ratio_NASPA_ = 50.8% vs. female ratio_Frambu_ = 59.5%; (χ2 (1) = 0.788, *p* = .354).

### Gastrointestinal complaints

Compared to controls, HSP more often experienced constipation and alternating constipation and diarrhea. Within the HSP sample women reported more nausea, bloating and alternating constipation and diarrhea than men. There was no difference in reported frequency of symptoms between patients with pure and complex HSP. See Table 1.

**Table 1:** Percentage of 108 HSP subjects reporting at least “much” gastrointestinal complaints compared to 46,293 controls

Symptom/severity	Sample	
	HSP	Controls
Heartburn/acid regurgitation	5.9%	7.3%
Diarrhea	2.9%	7.3%
Constipation	14.6%**	5.7%
Alternating obstipation/diarrhea	8.0%**^,a^	2.9%
Bloating	7.0%^a^	11.5%

*Note*. **difference between samples is significant at the *p* < .001-level. ^a^significant within HSP sample gender difference, women higher (*p* < .05)

### Fecal incontinence

Compared to controls, HSP more frequently reported problems with uncontrollable flatulence and stool leakage weekly or daily during the last month. Compared to controls, HSP were less frequently able to hold back stools for 15 min after first feeling urge to evacuate. Fecal incontinence affected daily life at least weekly in 30% of the HSP group as well as controls. Within the HSP sample, women reported less ability to hold back their stools. Pure HSP reported more uncontrollable flatulence compared to complex HSP. See Table 2.

**Table 2:** Frequency of fecal problems among 108 HSP subjects compared to 46,293 controls

Symptom	Sample	
	HSP	Controls
Uncontrollable flatulence		
Weekly	31.4%**	13.5%
Daily	16.2%**	5.2%
Stool leakage/faecal incontinence		
Weekly	8.7%**	2.5%
Daily	2.9%**	0.5%
Fecal incontinence affects daily life		
Weekly	17.5%	16.9%
Daily	7%	9.1%
Inability to hold back stool 15 min after first feeling urge to evacuate bowels**	38.5%**^,a^	13.9%

*Note*. **difference between samples is significant at the *p* < .001-level. ^a^significant within HSP sample gender difference, women higher (*p* < .05)

### Urinary symptoms

In the HSP sample, both frequent daytime urination and nocturia were reported more often than among controls. HSP confirmed more frequent urinary urgency and involuntary leakage compared to controls. More persons in the HSP sample reported leaking a large amount, and that frequently having to get up at night to urinate was a problem, compared to controls. Within the HSP sample, 53.8% had consulted a doctor because of a leakage problem, and 56.9% had received treatment. Most reported medical treatment (81.8%), followed by pelvic floor exercises (18.2%) and surgery (9.1%). Within the HSP sample, subjects with pure HSP reported more problems getting up at night to urinate and women more frequently reported stress urinary incontinence. A detailed summary of the main findings regarding the HSP sample compared to controls is presented in Table 3.

**Table 3:** Frequency of urinary problems among 108 HSP subjects compared to controls

Symptom	Sample	
	HSP	Controls
Frequency of daytime urination		
8–11 times	20.8%**	9.7%
> 11 times	6.6%**	1.0%
Frequency of nighttime urination		
> 4 times	5.7%**	0.7%
Frequency of nighttime urination experienced as a personal problem	14.9%**	2.8%
Sudden, compelling urge to urinate that is difficult to suppress at least several times a week	51.9%**	14.4%
Involuntary loss of urine	53.7%**	26.2%
In connection with sudden and strong urge to void	58.3%	53.6%
At least daily/nightly	30.5%**	12.7%
At least a “large” amount	14.8%**	3.8%
Experienced as personal problem	21.7%**	8%

*Note*. **difference between samples is significant at the *p* < .001-level

## Discussion

The present report is a survey of gastronintestinal complaints and fecal and urinary incontinence in a large sample of adults with HSP aged > 30 years recruited from a non-clinical setting. The study measures were drawn from HUNT3, a large population survey, enabling comparison of the HSP sample to population controls. Compared to controls, subjects with HSP experienced more constipation and alternate constipation/diarrhea. Contributing factors may be increased tone in pelvic floor muscles, use of medications, diet, and spending more time sitting and being immobile. In a previously published health survey of the same HSP sample as the current report, participants reported on average 9.0 h of sitting daily compared to 5.7 h among controls. Furthermore, 45% of the HSP sample reported to use a wheelchair outdoors [18]. Constipation may lead to alternating constipation/diarrhea. Constipation is also a known independent risk factor linked to urinary incontinence [20]. As diarrhea may cause FI, keeping stools solid helps to minimize the risk of FI [21].

In a systematic review in multiple sclerosis the reported prevalence of constipation and FI was 40% [22]. In a survey of patients with amyotrophic lateral sclerosis, urge incontinence and constipation, but not FI, was higher compared to healthy controls [23]. Recently, in a cohort of 56 patients with Friedreich’s ataxia, 80% reported lower urinary tract symptoms and 64% reported bowel symptoms [24]. While urinary urge symptoms are common in HSP, fecal urge is less described. Almost one-third of the HSP subjects in our study reported being unable to hold back stools 15 min after first feeling the urge to evacuate bowels. In an earlier study, 10 of 11 HSP patients had some degree of rectal urgency and decreased anal sphincter tone [15]. In a three-generation family with dominant inherited pure HSP, six of 15 persons reported mild sphincter disturbance [25]. Rectal urgency is often unpredictable, and causes much distress. About one-third of HSP subjects and controls answered that FI interfered with their daily life on a daily or weekly basis, indicating the severity of the problem in both groups. Persons who experience FI tend not to share their condition with others, including their health care providers [10, 21, 26]. In adult patients with Alzheimer’s disease and multiple sclerosis, FI and impaired bowel function has been shown to increase anxiety and reduce quality of life [10].

We identified some gender differences within the HSP sample. More women reported bloating and alternating constipation and diarrhea compared to men. Functional gastrointestinal disorders are generally reported to occur more often among women [27]. Within the HSP sample, women more frequently reported both urge FI and stress urinary incontinence. Stress urinary incontinence in women is known to be an independent risk factor for FI, also in the general population [21].

A large proportion of the HSP subjects in our study reported voiding dysfunction in the form of urinary urgency, daytime frequency and nocturua, in addition to incontinence. This is in accordance with previous studies [7–9, 28]. In our survey we found no significant difference in micturition function between the pure and the complex forms of HSP, which is in accordance with a previous study [9]. Urinary leakage was perceived more of a problem in the HSP group than among controls, and more than half of the HSP responders sought medical advice for involuntary loss of urine and had received treatment, indicating the severity of the problem. The most prevalent reported treatment was medications (82%). In an Estonian study of 49 persons with HSP, 35% used medication (oxybutynine) for incontinence [9].

The current survey has limitations. We rely on self-reported complaints, thus excluding the possibility of cross-referencing and validating data with medical records and physical examination. We do not know the exact response rate in the HSP sample, and it may be that those who experience a larger burden are more likely to respond. It is important to note, however, that the data presented herein are part of a larger survey [18]. Thus, we have no reason to believe that persons with more gastrointestinal and urinary complaints would be particularly motivated to respond. Cognitive deficits are known to exist among persons with HSP and may impact self-report accuracy and symptom description. Finally, we did not have access genetic data. Important differences may exist in the symptom areas reported herein depending on genetic subtypes. However, requiring genetic data may have reduced our sample size considerably, particularly older participants.

## Conclusion

Gastrointestinal complaints and FI in addition to voiding dysfunction affects a considerable number of adult persons with HSP recruited from a non-clinical setting. Health care professionals should actively enquire about symptoms to assess appropriate management interventions for their patients with HSP.

## Abbreviations

HSP: Hereditary spastic paraparesis
HUNT3: The Nord-Trøndelag health study, third wave
M: Mean
NASPA: The National Patients’ Association for Hereditary Ataxia and Spastic Paraplegia
SD: Standard deviation
